# Editorial: A Global Perspective on Vaccines: Priorities, Challenges and Online Information

**DOI:** 10.3389/fimmu.2019.02556

**Published:** 2019-11-12

**Authors:** Aldo Tagliabue, Luciana C. C. Leite, Odile Y. Leroy, Rino Rappuoli

**Affiliations:** ^1^Institute for Genetic and Biomedical Research, National Research Council, Monserrato, Italy; ^2^Instituto Butantan, Saõ Paulo, Brazil; ^3^European Vaccine Initiative, Heidelberg, Germany; ^4^GlaxoSmithKline, Siana, Italy; ^5^Toscana Life Sciences, Siena, Italy; ^6^Imperial College, London, United Kingdom

**Keywords:** vaccines, antimicrobial resistance, vaccine hesitancy, vaccine education, technological platforms

Vaccines are one of the most successful stories in global health. For 99.99% of mankind history, life expectancy has been <30 years, but in the last 300 years human life has increased by 55 years, of which 35 were gained in the last century. Vaccines are accountable for a significant part of this extraordinary result. Since the first successful vaccine for smallpox in 1796, vaccines for many other diseases have been generated, taking advantage of the developments of science. Smallpox has been totally eradicated, and polio could be soon eliminated. Many other effective vaccines have significantly reduced the incidence of diseases that have killed millions of people in the past. Paradoxically, the decreased impact of infectious diseases at the global level is making people think that vaccines are no longer necessary. One of the reasons behind the decline in vaccine confidence is that many people have become complacent, and we face now the phenomenon of vaccine hesitancy. Thus, we can say that vaccines are victims of their own success.

The topic “A global perspective on vaccines: priorities, challenges and online information” focuses on the most crucial issues in the vaccine field, with a view on the years to come.

Since infections travel the whole world with no borders, the war against microbes is a global one. In this context, the action of military forces in vaccine development (Ratto-Kim et al.) has been an important initiator, and the worldwide effort includes organizations that fight long-known diseases and, more challenging, emerging infections such as SARS, MERS, Ebola, and Zika (Marinho de Andrade Zanotto and Leite). The globalized modern way of life, with the increased number of travelers throughout continents, is aggravating the situation. It is extremely important to ensure preparedness and efficacy of vaccines leveraging on innovation.

**Research & Development** remain the core of the evolving field. The -omics revolution, combined with the power of the extremely fast-growing technologies that will rapidly involve artificial intelligence, is providing a large variety of new vaccine candidates. There is a great need for harmonization and simplification for the newly generated vaccines. To this end, new platforms are being developed that will allow for new generation vaccines with built-in adjuvanticity for preventing multiple infections in a single shot.

Safety and affordability are characteristics that must be ensured in modern vaccines. Affordability will allow for fast access to effective vaccines worldwide, in particular to populations in developing countries. In this view, promoting local manufacturing is a key element in vaccine affordability (Rey-Jurado et al.). Side effects must be minimized, including the mild ones, to ensure the full safety of preventive treatments. In parallel, the new vaccines should have the highest effectiveness in all populations, from people living in the clean industrialized world to the populations that are constantly facing large microbial burden in infection-endemic areas. In this view, information on the co-evolution of microbes with the human host, coming from genome-wide association studies, is opening new avenues to the understanding of the mechanisms of resistance to infections. Studying the resistant subjects will pose the basis to design unprecedented preventive and therapeutic approaches. Malaria is an important example for this approach and is offering the possibility to rethinking vaccine design (1, 2).

**Clinical trials** are already being designed to prove the feasibility of new technological approaches in naïve and previously primed subjects. The understanding of the mechanisms and timing of prime-boosting immunizations is important, also in light of the problems observed with a candidate Dengue vaccine (3). Some examples are provided in this topic (Rauch et al., Launay et al., Yao et al.).

In the last decades, a complex global health system against known and unknown infectious disease threats has arisen, encompassing various formal and informal networks of organizations that serve different stakeholders, have varying goals, modalities, resources, and accountability; operate at different regional levels (i.e., local, national, regional, or global); and cut across the public, private-for-profit, and private-not-for-profit sectors. Organizations such as the Bill and Melinda Gates Foundation, the Global Alliance for Vaccine Immunization, and the Coalition for Epidemic Preparedness Innovations are potent drivers that are transforming the vaccine world (Bloom and Cadarette).

Among the new global health challenges that we are facing, the most alarming is the growing inefficacy of antibiotics. The excessive and incorrect use of antibiotics has accelerated the generation of resistant pathogens, which in many cases show resistance to multiple drugs. The increasing inefficacy of current antibiotics is expected to cause 10 million deaths per year in the world by 2050 (Tagliabue and Rappuoli). Vaccines could be the solution to **Antimicrobial resistance** (AMR) and its impending death toll. Thus, AMR is revolutionizing vaccine R&D to the point that priorities are being re-evaluated, and vaccines are being developed for diseases that we have for long considered harmless because curable with antibiotics. Furthermore, scientists are exploring the potential protective role of vaccines beyond the classical induction of pathogen-specific adaptive humoral and cellular immunity. An increasing body of experimental data is now supporting the notion that vaccines can have non-specific protective effects (Uthayakumar et al.). The concept of innate immune memory is starting to be exploited for the design of personalized effective adjuvants in novel vaccination strategies.

The impressive scientific and technological advancements and the huge global health efforts need to be paralleled by worldwide initiatives in vaccine **Education**. Thus, the high-level training for present and future vaccinologists plays a fundamental role in broadening conceptual and applied knowledge in the vaccine field (Lambert and Podda). But vaccine education should not be limited to raising expert vaccinologists. It should also target the general public. As already mentioned, vaccine hesitancy is spreading and could become a major threat for vaccine effectiveness. Public perception and public acceptance will make the difference between successful control of infections and failure with consequent scourge propagation. The use of modern social media could be extremely important to spread and popularize the correct information, avoiding ideological and political exploitation (Arif et al.), thereby increasing acceptance and compliance. False information, such as the idea that vaccines cause autism, is difficult to eliminate, despite the solid epidemiological data against it (4). The impact of social media on our society is huge and rapidly transforming. But eliminating vaccine hesitancy is a moral imperative.

## Author Contributions

AT wrote the manuscript. LL, OL, and RR critically revised it.

### Conflict of Interest

LL is an employee of the Instituto Butantan, a government controlled institution engaged in vaccine development and trials. RR is head of the GSK Vaccine research and development. The remaining authors declare that the research was conducted in the absence of any commercial or financial relationships that could be construed as a potential conflict of interest.
